# *Late Elongated Hypocotyl* Positively Regulates Salt Stress Tolerance in *Medicago truncatula*

**DOI:** 10.3390/ijms24129948

**Published:** 2023-06-09

**Authors:** Zhichao Lu, Haiyang Liu, Yiming Kong, Lizhu Wen, Yang Zhao, Chuanen Zhou, Lu Han

**Affiliations:** 1 The Key Laboratory of Plant Development and Environmental Adaptation Biology, Ministry of Education, School of Life Sciences, Shandong University, Qingdao 266237, China; lzcsdhz@163.com (Z.L.); 15949537052@163.com (H.L.); wlizhu@163.com (L.W.); aimee.yangzhao@sdu.edu.cn (Y.Z.); czhou@sdu.edu.cn (C.Z.); 2 College of Life Sciences, Shandong Normal University, Jinan 250014, China; lck7love@163.com

**Keywords:** circadian clock, *MtLHY*, salt tolerance, *MtFLS*, flavonoids

## Abstract

Abiotic stress, such as drought, osmotic, and salinity stresses, seriously affects plant growth and crop production. Studying stress-resistant genes that enhance plant stress tolerance is an efficient way to facilitate the breeding of crop species with high stress tolerance. In this study, we reported that the core circadian clock component, the *LATE ELONGATED HYPOCOTYL* (*LHY*) orthologue *MtLHY*, plays a positive role in salt stress response in *Medicago truncatula*. The expression of *MtLHY* was induced by salt stress, and loss-of-function mutants of *MtLHY* were shown to be hypersensitive to salt treatment. However, overexpression of *MtLHY* improved salt stress tolerance through a higher accumulation of flavonoids. Consistently, exogenous flavonol application improved the salt stress tolerance in *M. truncatula*. Additionally, *MtLHY* was identified as a transcriptional activator of the flavonol synthase gene, *MtFLS*. Our findings revealed that *MtLHY* confers plant salt stress tolerance, at least by modulating the flavonoid biosynthesis pathway, which provides insight into salt stress tolerance that links the circadian clock with flavonoid biosynthesis.

## 1. Introduction

Plants have evolved intricate molecular systems and physiological strategies to cope with unfavorable environmental conditions, such as drought, osmotic stress, and salinity. In recent years, it has been discovered that the circadian clock system plays a crucial role in responding to abiotic stress [1,2]. As an endogenous time-keeping mechanism, the circadian clock components synchronize the developmental and physiological behaviors with external environmental cycles, providing plants with adaptive strategies to cope with environmental oscillations [1,2]. The circadian clock is composed of multiple transcriptional feedback regulatory loops that induce the transcription of numerous abiotic stress-associated genes, enabling plant adaptation and survival in unfavorable conditions [3,4]. For example, in Arabidopsis, the evening-phased clock component *GIGANTEA* (*GI*) regulates salt stress tolerance through the salt overly sensitive (SOS) pathway [5]. Another core circadian clock component, *EARLY FLOWERING3* (*ELF3*), enhances salt tolerance by post-transcriptionally promoting GI degradation and inhibiting the transcription of *PHYTOCHROME-INTERACTING FACTOR4* (*PIF4*) [6]. Additionally, two core components of the circadian clock, *CIRCADIAN CLOCK-ASSOCIATED1* (*CCA1*) and *LATE ELONGATED HYPOCOTYL* (*LHY*), directly bind to the promoter of *C-REPEAT BINDING FACTOR* (*CBF1*, *CBF2*, and *CBF3*) and activate their expression in response to low temperature [7]. In soybean, two homologous *LHY* pairs negatively control drought tolerance by repressing the abscisic acid responses [8]. In rice, the *LHY/CCA1* ortholog, *OsCCA1*, synchronously confers multiple abiotic stress tolerance by transcriptionally regulating ABA signaling [9]. However, the involvement of *MtLHY*, the *LHY/CCA1* ortholog in *Medicago truncatula*, in abiotic stress responses remains to be determined, despite the extensive research on nodulation and leaf movement mechanisms in *M. truncatula.* [10]. 

Soil salinity is a major limiting factor to plant growth and crop production in agriculture [11,12,13]. To cope with salt stress, plants have developed various mechanisms to improve salt tolerance, including osmoregulation, ionic balance, and antioxidant defense systems [14,15,16,17,18,19]. In response to salt stress, plants accumulate osmoprotectants, such as glycine, betaine, and proline to adjust osmotic pressure [14]. As excessive Na^+^ accumulation in plants leads to membrane damage due to oxidative stress, cells must maintain ionic balance, particularly Na^+^/K^+^ homeostasis, by regulating ion transporters to achieve a low cytoplasmic Na^+^/K^+^ ratio [14]. Reactive oxygen species (ROS), including hydroxyl radical (OH^−^), superoxide anion (O_2_^−^), and hydrogen peroxide (H_2_O_2_) are highly reactive molecules that are generally induced in response to abiotic stress [20]. Excessive ROS accumulation leads to oxidative stress, causing membrane damage and even cell death [20,21,22]. Therefore, plants activate antioxidant protection systems to remove excessive ROS and protect the membrane structure. ROS detoxification is regulated by cytosolic enzymatic antioxidants, including peroxidase (POD), catalase (CAT), and superoxide dismutase (SOD) [23]. Additionally, excessive ROS can be eliminated by nonenzymatic antioxidants, such as glutathione (GSH), alkaloids, carotenoids, and flavonoids [24]. 

Flavonoids are a major group of secondary metabolites found in most plants and their organs. They are known for their ability to scavenge ROS efficiently and are divided into various subgroups, including chalcones, flavones, flavonols, and isoflavones [25,26,27]. Recently, there has been increasing interest in the role of flavonoids in protecting plants against environmental stresses such as UV radiation, cold, drought, and salinity [26,28,29,30]. For example, in the pigeon pea, the CcCIPK14-CcCBL1 complex regulates flavonoid biosynthesis and plays a crucial role in drought stress tolerance [31]. In soybean, silencing of the flavone synthase gene, *GmFNSII*, resulted in reduced flavone content and hypersensitivity to salt treatment in hairy roots [32]. Heat shock factor *HSFB2b* inhibited the expression of *GmNACs* to promote flavonoid biosynthesis, which conferred soybean salt stress tolerance [33]. Additionally, exogenous flavonoid application reduced MDA content and increased salt stress tolerance [33]. Therefore, flavonoid accumulation is strongly associated with salt stress responses. 

In this study, we discovered the core component of the circadian clock, *MtLHY*, is responsible for salt stress tolerance in *M. truncatula*. Loss of function in *MtLHY* results in a hypersensitivity phenotype to salt treatment, while the ectopic expression of *MtLHY* increases the ability of salt stress tolerance in *M. truncatula. MtLHY* plays a crucial role in inhibiting ROS production and mediating Na^+^ /K^+^ homeostasis under salt stress. Additionally, we found that *MtLHY* plays a key role in modulating flavonoid biosynthesis. *MtLHY* directly binds to the promoters of *MtFLS* to activate its expression, and a high content of flavonols accumulates in *MtLHY*-overexpressing plants to resist salt stress. Consistently, exogenous flavonols application also enhances the salt stress tolerance in *M. truncatula.* Thus, our results support the role of *MtLHY* as a positive regulator in salt stress tolerance, linking the circadian clock and flavonoid biosynthesis to coordinate plant growth and environmental adaptation.

## 2. Results

### 2.1. Loss-of-Function Mutants of MtLHY Is Hypersensitive to Salt Stress

**Figure 1:**
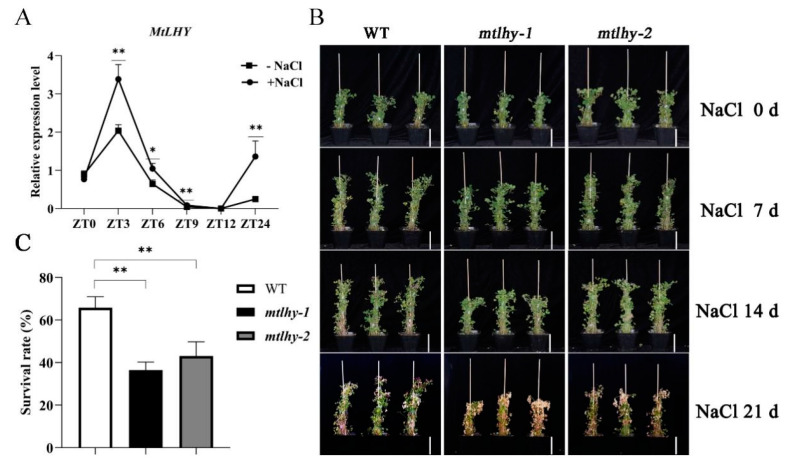
Loss-of-function mutants of *MtLHY* were shown to be hypersensitive to salt treatment: The expression of *MtLHY* was induced by salt stress (**A**). Loss-of-function mutants of *MtLHY* were shown to be hypersensitive to salt treatment with 150 mM NaCl for 3 weeks (**B**). Statistical analysis of the survival rate of WT and *mtlhy* mutants at treatment with 150 mM NaCl for 3 weeks (**C**). Data are shown as means ± SD (*n* = 15; * p < 0.05, ** *p* < 0.01, based on Dunnett test). Scale bar, 10 cm.

Previous research has demonstrated that *LHY* orthologues are a crucial component of the circadian clock and play a vital role in plant abiotic stress responses, such as drought and cold stress [7,8]. However, there is limited information regarding the involvement of *LHY* in response to salt stress. To investigate the role of *LHY* in the response to salinity stress in *M. truncatula,* we initially examined the expression patterns of *MtLHY* in response to salt treatment. The results show that the transcript of *MtLHY* could be rapidly induced by the salt stress treatment. Its transcript level was increased by only 3 h (hours) after treatment with 150 mM NaCl and remained at higher levels for 9 h more than the control without salt treatment (Figure 1A), suggesting it may be involved in response to salt stress. 

To further investigate whether *MtLHY* plays a key role in salt tolerance, we screened and obtain two independent *Tnt1* insertion mutants of *MtLHY* from the *Tnt1*-tagged mutant population [10]. We then subjected 4-week-old *mtlhy* mutants and wild-type (WT) plants to salt stress treatments and analyzed their survival rates after treatment with 150 mM NaCl for 3 weeks (Figure 1B,C). Our statistical analysis showed that approximately 65.9% of WT plants survived, while only 36.5–43.1% of the two *mtlhy* alleles recovered to live (Figure 1C), suggesting the loss of function of *MtLHY* resulted in a hypersensitivity phenotype to salt stress.

### 2.2. MtLHY Mutation Impaired Seed Germination and Seedling Growth under NaCl Stress

**Figure 2:**
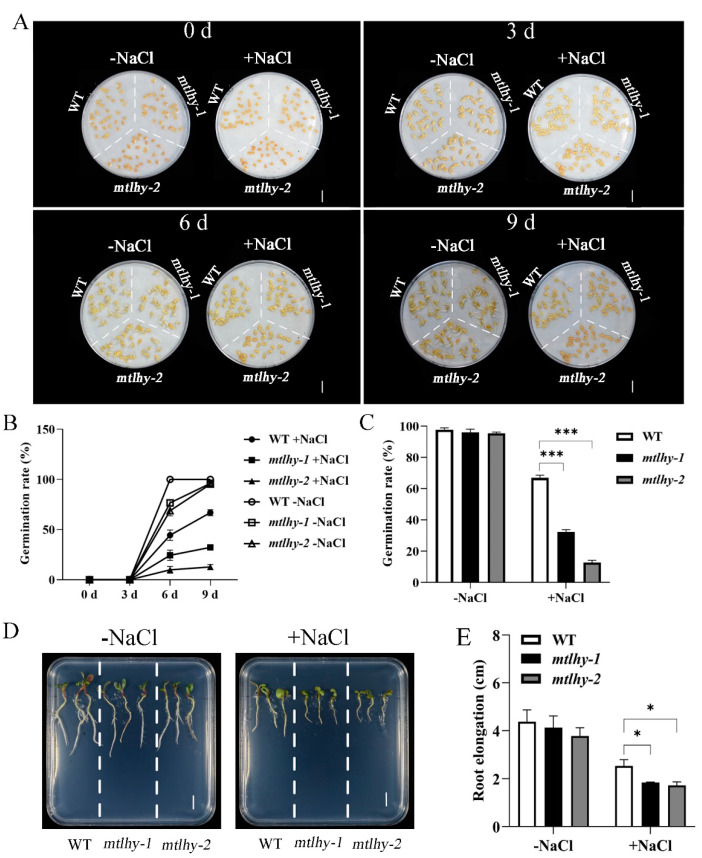
*MtLHY* mutation impaired seed germination and seedling growth under NaCl stress: Seed germination phenotype of *mtlhy* mutants and WT with or without a 150 mM NaCl treatment (**A**). Statistical analysis of seed germination rate of *mtlhy* mutants and WT with or without a 150 mM NaCl treatment (**B**,**C**). The seedling phenotype of *mtlhy* mutants and WT with or without a 150 mM NaCl treatment (**D**). Statistical analysis of root length of *mtlhy* mutants and WT with or without a 150 mM NaCl treatment (**E**). Data are shown as means ± SD (*n* = 3; * *p* < 0.05, *** *p* < 0.001, based on Dunnett test). Scale bar, 1 cm.

Salt is a crucial factor that impacts seed germination and seedling growth [34]. To confirm the involvement of *MtLHY* in these processes under salt stress, we studied the phenotype of *mtlhy* mutants and WT with or without a 150 mM NaCl treatment. Firstly, we tested the seed germination phenotype of mutants and WT (Figure 2A). The WT seeds sprouted completely on the sixth day without treatment, but the *mtlhy* mutants required 9 d (days) (Figure 2A,B), indicating that the loss of function of *MtLHY* delayed seed germination. After treatment with 150 mM NaCl, salt stress significantly inhibited seed germination rates in both *mtlhy* mutants and WT (Figure 2A–C). In particular, the mutants exhibited significantly lower seed germination rates than WT seeds after NaCl treatment for 9 d (Figure 2A–C). For example, the germination rate of WT seeds was approximately 66.9% at 150 mM NaCl, whereas the mutants were 12.8–32.3% at that point (Figure 2C).

Root growth was more affected by salt stress than shoot growth [35]. To further identify the function of *MtLHY* on the postgermination seedling under salt stress, we performed experiments to measure root length in both WT and *mtlhy* mutant seedlings. When grown on ½MS medium, both WT and *mtlhy* mutant seedlings exhibited similar root lengths (Figure 2D,E). However, the root length of the mutant seeding was significantly shorter than that of WT at 150 mM NaCl for a week (Figure 2D,E). These findings collectively suggest that *MtLHY* plays a crucial role in seed germination and seedling growth under salt stress conditions. 

### 2.3. Physiological Responses of MtLHY Mutation to Salt Stress

**Figure 3:**
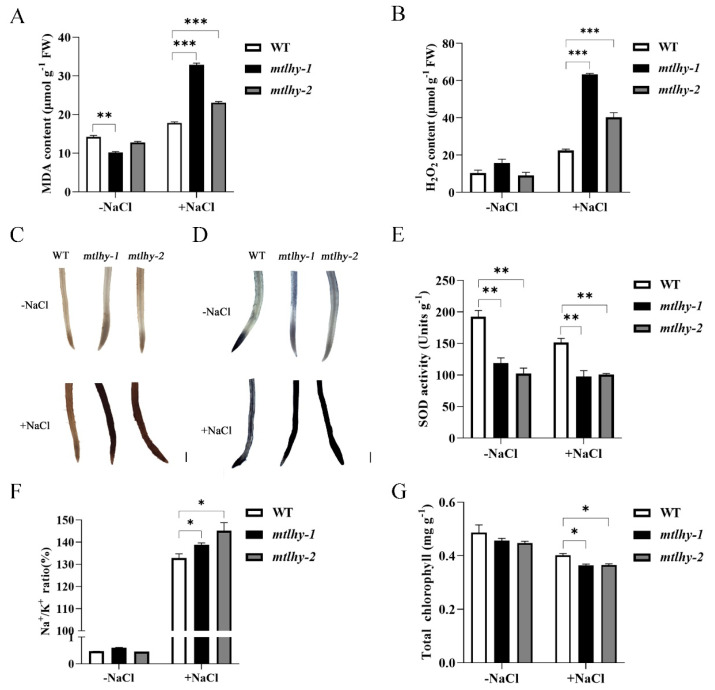
Physiological responses of *mtlhy* mutant to salt stress: The concentrations of MDA were determined in the leaves of *mtlhy* mutants and WT with or without a 150 mM NaCl treatment for 7 d (**A**). The content of H_2_O_2_ was determined in the leaves of *mtlhy* mutants and WT with or without a 150 mM NaCl treatment for 7 d (**B**). The accumulation of hydrogen peroxide (H_2_O_2_) in the root of *mtlhy* mutants and WT was visualized by staining with 3,3’-diaminobenzidine (DAB) with or without a 150 mM NaCl treatment for 7 d (**C**). The accumulation of superoxide anion (O_2_^−^) in the root of *mtlhy* mutants and WT was visualized by staining with nitroblue tetrazolium (NBT) with or without a 150 mM NaCl treatment for 7 d (**D**). SOD activity was examined in the leaves of *mtlhy* mutants and WT with or without a 150 mM NaCl treatment for 7 d (**E**). Na^+^/K^+^ ratio and total chlorophyll content were examined in the leaves of *mtlhy* mutants and WT with or without a 150 mM NaCl treatment for 7 d (**F**,**G**). Data are shown as means ± SD (*n* = 3; * *p* < 0.05, ** *p* < 0.01 *** *p* < 0.001, based on Dunnett test). Scale bar, 1 mm.

To assess the physiological response of *mtlhy* mutants and WT to salt stress, we initially compared the levels of malondialdehyde (MDA), which is an indicator of lipid peroxidation [36]. Our findings showed that *mtlhy* mutant leaves had a higher accumulation of MDA than WT at 150 mM NaCl (Figure 3A) for a week, suggesting that the loss of function of *MtLHY* resulted in increased cell membrane damage in response to NaCl stress. Subsequently, we observed that the production of H_2_O_2_ in *mtlhy* leaves was significantly higher than in WT leaves under NaCl stress (Figure 3B), indicating high levels of ROS in the mutants. This was further confirmed by both 3,3’-diaminobenzidine (DAB) and nitroblue tetrazolium (NBT) staining in roots (Figure 3C,D). While the rapid accumulation of ROS in plant cells is an effective strategy in response to stress, elevated ROS levels can be harmful to the plant [37]. Therefore, the balance between ROS production and detoxification is crucial for plant tolerance to stress. Notably, the reduced magnitude of SOD activity in the *mtlhy* mutants indicates that the terms of ROS detoxification were impaired in the *mtlhy* mutants (Figure 3E). As Na^+^/K^+^ homeostasis in the cytoplasm is critical for salt tolerance, and a low Na^+^/K^+^ ratio is an indicator of salt tolerance [38], we analyzed the Na^+^/K^+^ ratio in the WT leaves and *mtlhy* mutants subjected to salt treatment. The Na^+^/K^+^ ratio of *mtlhy* leaves was significantly higher than that of WT (Figure 3F). Conversely, the chlorophyll content of *mtlhy* leaves was lower than that of WT under salt stress (Figure 3G). These data collectively indicated that *mtlhy* mutants were hypersensitive to salt stress.

### 2.4. MtLHY Overexpression Improves Salt Stress Tolerance in M. truncatula

**Figure 4:**
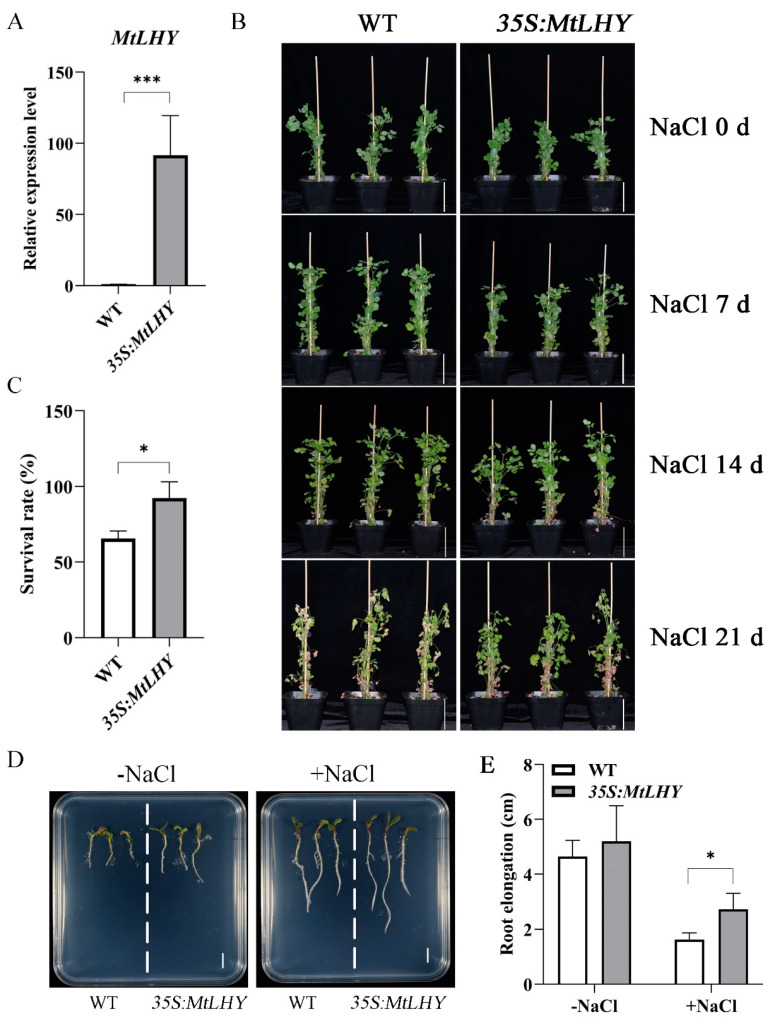
*MtLHY* overexpression improves salt stress tolerance in *M. truncatula*: The expression level of *MtLHY* in *MtLHY*-overexpressing plants (**A**). *MtLHY*-overexpressing plants were shown to be resistant to salt treatment with 150 mM NaCl for 3 weeks (**B**). Statistical analysis of the survival rate of WT and *MtLHY*-overexpressing plants at treatment with 150 mM NaCl for 3 weeks (**C**). The seedling phenotype of *MtLHY*-overexpressing plants and WT with or without a 150 mM NaCl treatment (**D**). Statistical analysis of root length of *MtLHY*-overexpressing plants and WT with or without a 150 mM NaCl treatment (**E**). Data are shown as means ± SD (*n* = 3; * *p* < 0.05, *** *p* < 0.001, based on two-tailed *t*-tests). Scale bars, 10 cm in (**B**) and 1 cm in (**D**).

To further investigate the role of *MtLHY* in regulating salt stress tolerance in *M. truncatula*, we generated transgenic plants overexpressing *MtLHY*, resulting in significantly elevated transcript levels of *MtLHY* in leaves (Figure 4A). These plants were selected for further study to explore the salt stress tolerance conferred by *MtLHY* expression. *MtLHY*-overexpressing transgenic plants and WT plants were subjected to salt treatments (Figure 4B), and statistical data showed that the survival rate of WT plants was approximately 65.6% at 150 mM NaCl for three weeks (Figure 4C), while that of *MtLHY*-overexpressing lines was over 92.3% (Figure 4C), indicating that elevated *MtLHY* transcript levels enhanced salt stress tolerance in *M. truncatula.* Additionally, we measured the root lengths of seedlings from both the overexpression plants and WT grown on ½MS medium with or without 150 mM NaCl treatment for a week (Figure 4D). Under normal conditions, the root lengths of *MtLHY*-overexpressing seedlings were comparable to those of WT (Figure 4D,E). However, in the presence of salt stress, the root lengths of *MtLHY*-overexpressing transgenic lines were significantly longer than those of WT (Figure 4D,E), indicating that *MtLHY* overexpression promotes efficient seedling growth under salt stress conditions. Overall, our results suggest that *MtLHY* overexpression improves salt stress tolerance in *M. truncatula*.

**Figure 5:**
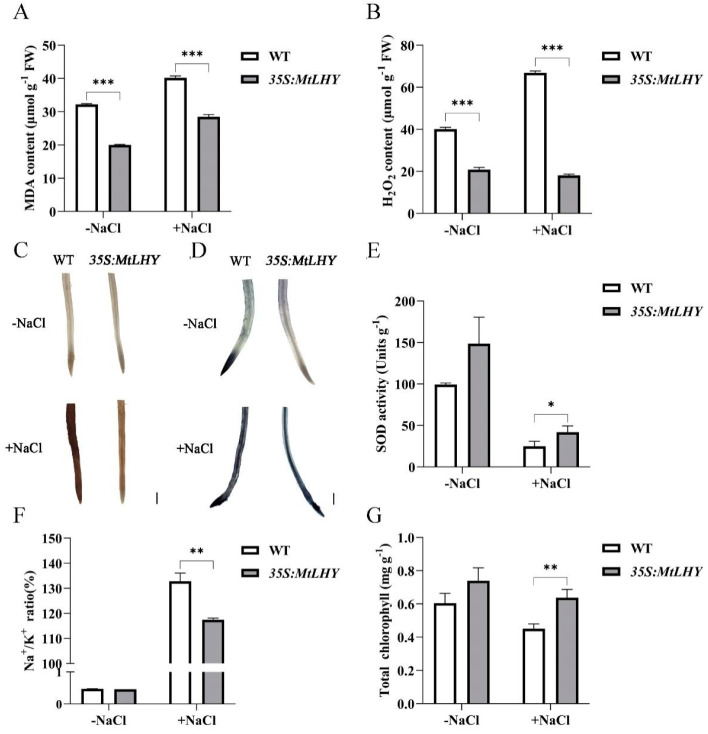
Physiological responses of *MtLHY*-overexpressing plants to salt stress: The concentrations of MDA were determined in the leaves of *MtLHY*-overexpressing plants and WT with or without a 150 mM NaCl treatment for 7 d (**A**). The content of H_2_O_2_ was determined in the leaves of *MtLHY*-overexpressing plants and WT with or without a 150 mM NaCl treatment for 7 d (**B**). The accumulation of hydrogen peroxide (H_2_O_2_) in the root of *MtLHY*-overexpressing plants and WT was visualized by staining with 3,3′-diaminobenzidine (DAB) with or without a 150 mM NaCl treatment for 7 d (**C**). The accumulation of superoxide anion (O_2_^−^) in the root of *MtLHY*-overexpressing plants and WT was visualized by staining with nitroblue tetrazolium (NBT) with or without a 150 mM NaCl treatment for 7 d (**D**). SOD activity was examined in the leaves of *MtLHY*-overexpressing plants and WT with or without a 150 mM NaCl treatment for 7 d (**E**). Na^+^/K^+^ ratio and total chlorophyll content were examined in the leaves of *MtLHY*-overexpressing plants and WT with or without a 150 mM NaCl treatment for 7 d (**F**,**G**). Data are shown as means ± SD (*n* = 3; * *p* < 0.05, ** *p* < 0.01 *** *p* < 0.001, based on two-tailed *t*-tests). Scale bar, 1 mm.

To further validate our findings, we examined the physiological responses of *MtLHY*-overexpressing plants to NaCl stress. Compared with the WT plants, *MtLHY*-overexpressing plants exhibited lower levels of MDA accumulation (Figure 5A), regardless of whether they were treated with 150 mM NaCl or not. Additionally, the H_2_O_2_ content in *MtLHY*-overexpressing plants was significantly lower than that in WT (Figure 5B), indicating that high levels of *MtLHY* expression enhance the capacity for ROS scavenging. This was further confirmed by DAB and NBT staining in roots (Figure 5C,D). Moreover, *MtLHY* overexpression increased SOD activity under salt conditions (Figure 5E), suggesting that *MtLHY* plays a key role in regulating SOD activity. Notably, the Na^+^/K^+^ ratio in the leaves of *MtLHY*-overexpressing lines was lower than that in WT (Figure 5F), while the chlorophyll content in the leaves of *MtLHY*-overexpressing plants was higher than that in WT (Figure 5G), suggesting that *MtLHY* mediates Na^+^/K^+^ homeostasis in response to salt stress. In summary, *MtLHY* plays a positive role in salt tolerance.

### 2.5. MtLHY Is Involved in Flavonoid Biosynthesis

**Figure 6:**
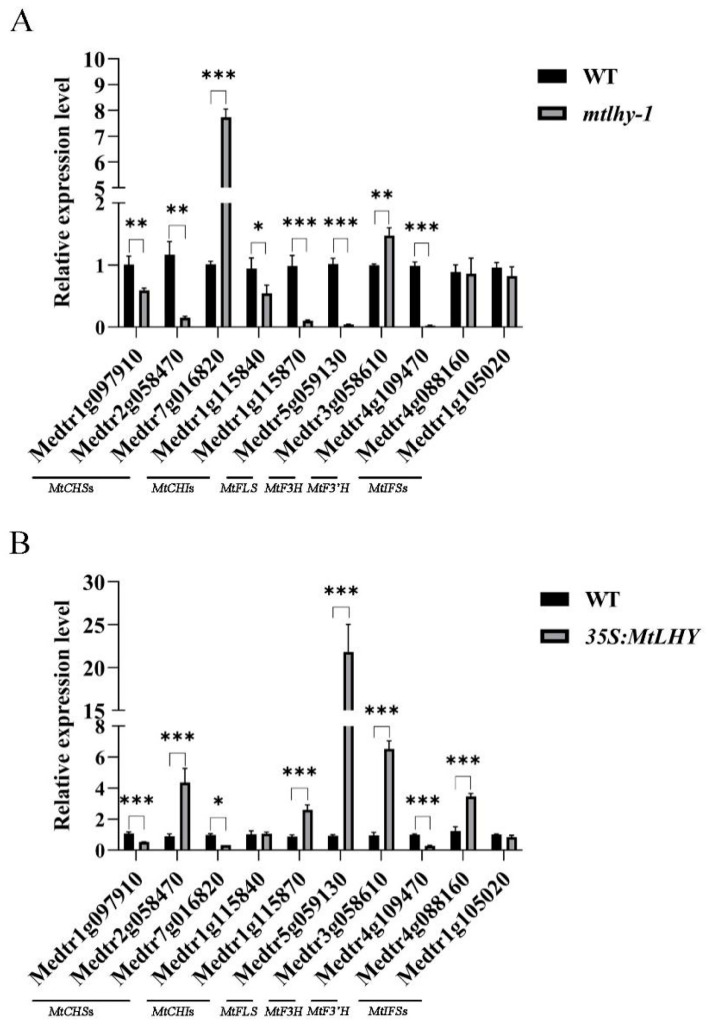
*MtLHY* is involved in flavonoid biosynthesis: The transcript levels of known flavonoid biosynthesis genes, such as *MtCHS*, *MtCHI*, *MtFLS*, *MtF3*, *MtF3’H*, and *MtIFS*, were examined in both *mtlhy* mutants and *MtLHY*-overexpressing plants compared with those in WT (**A**,**B**). Data are shown as means ± SD (*n* = 3; * *p* < 0.05, ** *p* < 0.01 *** *p* < 0.001, based on two-tailed *t*-tests).

To further identify the direct targets of *MtLHY* in transcriptional regulation, we compared the differentially expressed genes (DEGs) in the leaves of WT and *mtlhy* mutants from our previous RNA-sequencing transcriptome data [10]. Kyoto Encyclopedia of Genes and Genomes (KEGG) analysis revealed that 63 DEGs were enriched in the flavonoid biosynthesis pathway, which is one of the top biological processes. Flavonoid compounds have been shown to possess antioxidant activity that can prevent damage caused by free radicals by scavenging ROS, activating antioxidant enzymes, and inhibiting NADPH oxidases. To investigate whether *MtLHY* is involved in flavonoid biosynthesis, we selected key enzyme genes involved in flavonoid biosynthesis from the DEGs and verified their expression levels using RT-qPCR in both WT and *mtlhy* mutant plants, as well as in *MtLHY*-overexpressing plants. The transcript levels of known flavonoid biosynthesis genes, such as *MtCHS*, *MtCHI*, *MtFLS*, *MtF3*, *MtF3’H*, and *MtIFS*, were significantly altered in both *mtlhy* mutants and *MtLHY*-overexpressing plants compared with those in WT (Figure 6). Notably, *MtFLS*, the gene responsible for flavonol synthase, was significantly downregulated in *mtlhy* mutants but significantly upregulated in *MtLHY*-overexpressing plants (Figure 6), suggesting that the expression of *MtFLS* is likely induced by MtLHY. 

### 2.6. MtLHY Overexpression Enhances Flavonoid Accumulation

**Figure 7:**
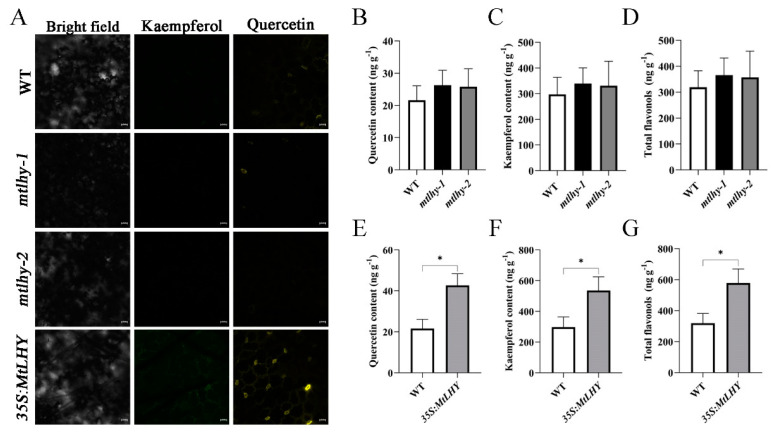
*MtLHY* overexpression enhances flavonoid accumulation in leaves: Significant DPBA fluorescence signals were detected in the guard cells of *MtLHY*-overexpressing plants (**A**). The content of quercetin (**B**), kaempferol (**C**), and total flavonoids (**D**) was examined in the leaves of *mtlhy* mutants and WT plants under a 150 mM NaCl treatment for 7 d (**B**–**D**); data are shown as means ± SD (*n* = 3; based on Dunnett test). The content of quercetin (**E**), kaempferol (**F**), and total flavonoids (**G**) was examined in the leaves of *MtLHY*-overexpressing plants and WT plants under a 150 mM NaCl treatment for 7 d (**E**–**G**); data are shown as means ± SD (*n* = 3; * *p* < 0.05, based on two-tailed *t*-tests). Scale bar, 20 μm.

As we know, *FLS* encodes flavonol synthase that catalyzes the formation of flavonols from dihydroflavonols. Based on the fact that *MtFLS* is likely induced by *MtLHY*, we hypothesize that *MtLHY* plays a crucial role in flavonol biosynthesis. To test this hypothesis, we investigated the flavonol content in WT, *MtLHY*, and *MtLHY*-overexpressing plants. We used diphenylboric acid 2-aminoethyl ester (DPBA) to image flavonol accumulation in leaves, as DPBA is a fluorescent dye that binds specifically to two flavonols, kaempferol and quercetin [39]. Significant DPBA fluorescence signals were detected in the guard cells of *MtLHY*-overexpressing plants (Figure 7A). However, weak DPBA fluorescence signals were observed in both WT and *mtlhy* mutants, with no significant difference (Figure 7A). These data suggest that overexpression of *MtLHY* leads to flavonol accumulation in guard cells. To demonstrate changes in flavonol and flavonoid abundance under salt stress, we performed high-pressure liquid chromatography–mass spectroscopy (LC-MS) to quantify flavonol and total flavonoids concentrations in WT, *mtlhy* mutants, and *MtLHY*-overexpressing plant leaves. We found no difference in the content of total flavonoids between WT and *mtlhy* mutants (Figure 7D). The two flavonol components, kaempferol and quercetin, also showed no difference between WT and *mtlhy* mutants (Figure 7B,C), indicating that the content of both kaempferol and quercetin in *mtlhy* mutants is not the primary factor causing the hypersensitivity phenotype to salt stress. However, the content of total flavonoids, including kaempferol and quercetin, was significantly higher in the *MtLHY*-overexpressing plant compared with WT (Figure 7E–G). Taken together, overexpression of *MtLHY* enhances the accumulation of flavonoids, including kaempferol and quercetin. 

### 2.7. Application of Exogenous Flavonols Enhances Salt Stress Tolerance in M. truncatula

**Figure 8:**
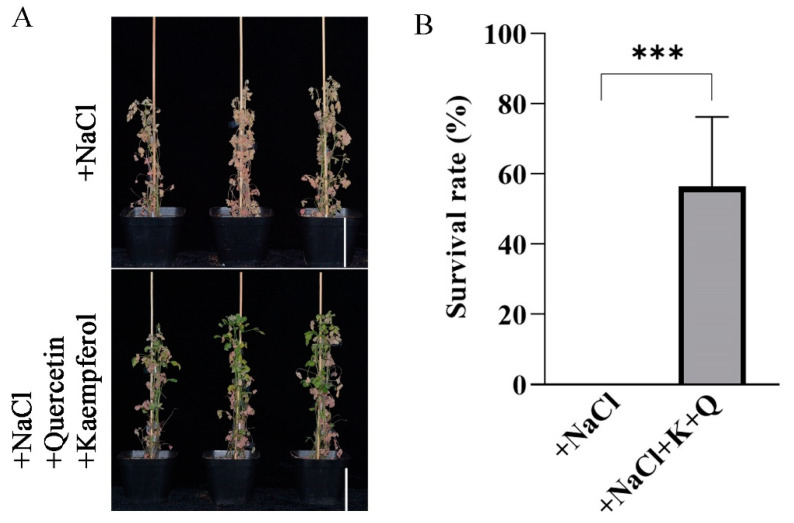
Application of exogenous flavonols enhances salt stress tolerance in *M. truncatula*: Application treatment of WT plants with exogenous 15 μm kaempferol and 15 μm quercetin in the Hoagland nutrient solution under 150 mM salt stress conditions. Kaempferol and quercetin were dissolved in ethanol separately, and the control sample was treated with an equal volume of ethanol (**A**). Under salt stress conditions, statistical analysis of the survival rate of the WT plants with or without the exogenous application of kaempferol (K) and quercetin (Q) (**B**). Data are shown as means ± SD (*n* = 3; *** *p* < 0.001, based on two-tailed *t*-tests). Scale bar, 10 cm.

Based on the known ability of flavonols to prevent cellular damage caused by salt stress and the fact that overexpression of *MtLHY* can increase flavonol accumulation, we hypothesize that high levels of flavonols can enhance salt stress tolerance in *M. truncatula*. Previous studies have shown that exogenous flavonoids can confer salt stress tolerance in soybean [33]. To investigate whether flavonols play a similar role in salt stress tolerance in *M. truncatula*, we treated WT plants with exogenous kaempferol and quercetin under salt stress conditions (Figure 8A). After 40 d, we assessed the survival rate of plants with and without the application of exogenous flavonols. Statistical analysis revealed that approximately 56.3% of WT plants survived with the application of exogenous flavonols at 150 mM NaCl, while all plants without the application died (Figure 8B). Therefore, the application of exogenous flavonols enhances salt tolerance in *M. truncatula*. 

### 2.8. MtLHY Transcriptionally Activates MtFLS 

**Figure 9:**
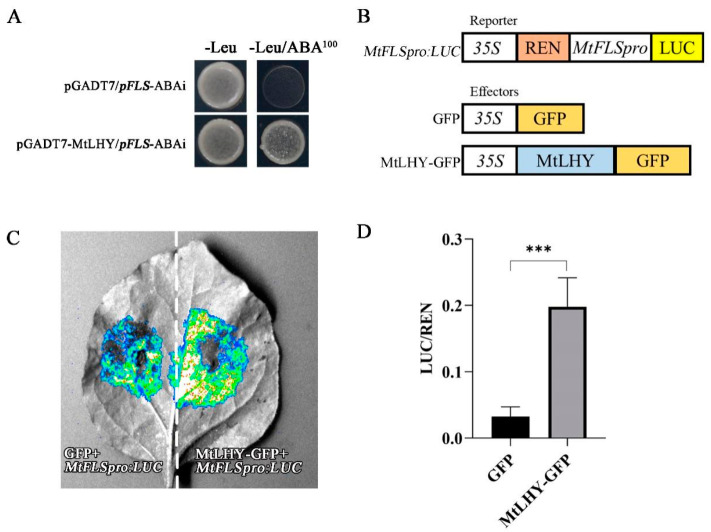
MtLHY transcriptionally activates *MtFLS*: A yeast one-hybrid (Y1H) assay showed that the *MtLHY* protein binds to the promoter sequences of *MtFLS* (**A**). Schematic representations of the reporter and effector constructs were used in the transient expression assay (**B**). A dual luciferase assay showed MtLHY increased the expression of LUC driven by the promoter of *MtFLS* (**C**,**D**). Data are shown as means ± SD (*n* = 4; *** *p* < 0.001, based on two-tailed *t*-tests).

As the expression of *MtFLS* was found to be regulated by MtLHY, we hypothesize that *MtFLS* is a potential direct target of MtLHY. To test this hypothesis, we performed a yeast one-hybrid (Y1H) experiment, which demonstrated that the MtLHY protein binds to the promoter sequences of *MtFLS* (Figure 9A). Subsequently, we performed a dual luciferase assay to investigate the effects of MtLHY on the promoter of *MtFLS*. The results indicate that MtLHY increased the expression of LUC driven by the promoter of *MtFLS* (Figure 9B,C). Therefore, MtLHY transcriptionally activates the expression of *MtFLS*.

## 3. Discussion

**Figure 10:**
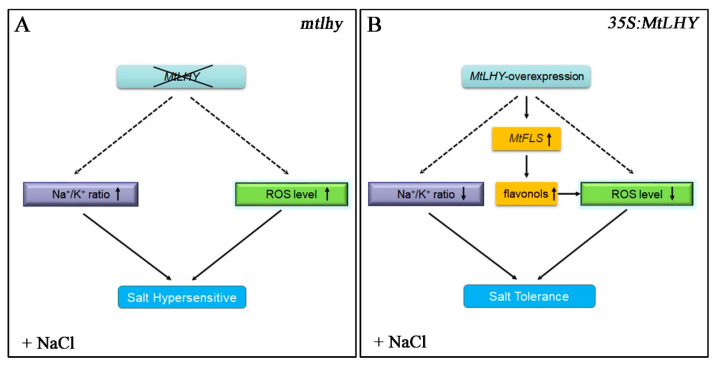
A proposed working model for *mtlhy* mutants and *MtLHY*-overexpressing plants under salt stress: A proposed working model for *mtlhy* mutants under salt stress (**A**). A proposed working model for *MtLHY*-overexpressing plants under salt stress (**B**). The dotted arrow means the indirect ation and the solid arrow means the direct ation.

Soil salinization is a global issue that is becoming increasingly severe. Improper fertilization, insufficient irrigation, and the intrusion of seawater all contribute to the accumulation of salt in the soil, which impairs plant growth [40]. Thus, the most effective and ultimate solution to cope with salt stress is to utilize salt-resistant genes to cultivate high-resistance species. In this study, we proposed a working model in which the core component of the circadian clock, *MtLHY*, plays a positive role in salt stress tolerance by regulating ROS homeostasis, Na^+^/K^+^ homeostasis, and flavonoid accumulation (Figure 10). Our findings suggested that the circadian clock may serve as a potential target for improving salt stress tolerance in crops.

ROS are induced by abiotic stress, and their excessive accumulation leads to membrane oxidation, DNA damage, and even cell death [20,21,22]. In recent years, it has been shown that *LHY/CCA1* orthologs inhibit the production of ROS [9,41]. In Arabidopsis, overexpression of *CCA1* has been found to enhance tolerance to oxidative stress, while a mutation in *CCA1* results in a hypersensitive phenotype [41]. In rice, loss-of-function mutants of *OsCCA1/OsLHY* accumulate high levels of ROS when subjected to salt treatment [9]. Our study found that *MtLHY*-overexpressing plants have a lower ROS level than WTs under salt stress, while *mtlhy* mutants have a higher ROS level, indicating a conserved role of *LHY/CCA1* orthologs in maintaining ROS homeostasis to cope with abiotic stress. However, the function of *LHY/CCA1* orthologs in abiotic stress has been subdivided and neofunctionalized during seed plant evolution. For example, mutations in two homologous pairs of *LHY*, *GmLHY1a* and *GmLHY1b*, have been found to confer drought tolerance in soybeans [8]. On the other hand, mutations in the other two homologous pairs, *GmLHY2a* and *GmLHY2b*, are still sensitive to drought, indicating the different roles of the involvement of *GmLHY1* and *GmLHY2* in drought tolerance. Interestingly, overexpression of *GmLHY1a* and *GmLHY1b* enhances abscisic acid resistance in both Arabidopsis and soybean, suggesting the conserved role of *GmLHY1a* and *GmLHY1b* in regulating the ABA signaling pathway [8]. Conversely, loss-of-function mutants of *OsCCA1/OsLHY* are hypersensitive to drought stress, indicating that the role of *LHY* orthologs involved in drought stress depends on the specific species. However, the biological functions of *LHY* orthologs involved in salt stress are conserved in rice and *M. truncatula*. Overall, the biological function of *LHY/CCA1* orthologs involved in abiotic stress has been subdivided and neofunctionalized, depending on the specific species, types of abiotic stresses, or their respective downstream targets. 

*MtLHY*, a member of the MYB transcription factor family, is postulated to exert its biological functions by modulating the expression of target genes. Our RNA-seq data revealed that *MtLHY* plays a role in regulating genes involved in flavonoid biosynthesis, specifically by directly activating the expression of *MtFLS*, a flavonol synthase gene. Subsequently, overexpression of *MtLHY* resulted in an increased accumulation of flavonoids in *M. truncatula*. Flavonoids, known for their efficient ROS scavenging properties, have been extensively studied in recent years. In plants, flavonoids have been demonstrated to play a protective role against damage caused by biotic and abiotic stresses. We observed a decrease in ROS levels in the *MtLHY*-overexpressing plant under salt treatment, indicating that *MtLHY* overexpression may enhance salt tolerance by increasing the accumulation of flavonoids, particularly kaempferol and quercetin, in leaves. This prediction is supported by the fact that the application of kaempferol and quercetin has been shown to improve plant survival under salt stress. Therefore, it is likely that *MtLHY* overexpression improves salt tolerance, at least in part, through the antioxidant activity of flavonols. In addition, the application of quercetin has a similar effect on soybean under salt stress [33], indicating the critical role of flavonols in enhancing leguminous salt tolerance. Unfortunately, the levels of both kaempferol and quercetin in *mtlhy* mutants have not changed, suggesting that their contents are not responsible for the hypersensitive phenotype of the mutants. Currently, there are two hypotheses proposed to explain this observation. One hypothesis posits that other MtFLS orthologous or homologous proteins have redundant roles in flavonol biosynthesis. In *M. truncatula*, there are four orthologous of MtFLS, with unknown functions. MtFLS orthologous proteins likely play a redundant role in controlling the biosynthesis of kaempferol and quercetin to maintain their levels. The alternative hypothesis suggests that other flavonol compounds may be responsible for the hypersensitive phenotype of mutants. Flavanols, a subgroup of flavonoids, include compounds such as kaempferol, quercetin, fisetin, and myricetin. Other types of flavonols may be responsible for the hypersensitive phenotype of mutants. Furthermore, based on our data, we hypothesize that *MtLHY* contributes to plant salt tolerance by regulating various pathways, including stress-related signaling pathways, scavenging of ROS, and osmotic adjustment. Performing RNA-seq, DNA affinity purification sequencing (DAP-seq), and chromatin immunoprecipitation sequencing (ChIP-seq) experiments under salt stress conditions will enable us to investigate this hypothesis and identify the direct targets of *MtLHY* involved in controlling salt stress response on a genome-wide scale. Additionally, *MtFLS* is directly activated by the expression of *MtLHY*, and the application of exogenous flavonols has been shown to enhance the survival of *Medicago* plants under salt stress, indicating the crucial involvement of flavonol synthase genes in salt stress response. In *Arabidopsis thaliana*, ectopic expression of *DoFLS1*, a flavonol synthase gene from *Dendrobium officinale*, leads to an increase in flavonol production and improved tolerance to abiotic stress [42]. Ectopic expression of *EkFLS* from *Euphorbia kansui Liou* in Arabidopsis promotes flavonoid accumulation, significantly enhancing the activities of POD and SOD, which in turn improves abiotic stress tolerance in plants [42]. Furthermore, overexpression of an *Apocynum venetum* flavonol synthase gene has been found to enhance salinity stress tolerance in transgenic tobacco plants [43]. Therefore, these findings suggest that overexpression of flavonol synthase genes may be a promising strategy for improving plant tolerance to salt stress. Characterization of the loss-of-function *mtfls* mutants and *MtFLS*-overexpression plants under salt stress in *M. truncatula* could provide insights into the functions of *MtFLS* in the response to salt stress.

## 4. Materials and Methods

### 4.1. Plant Materials and Growth Environments

*Medicago truncatula* ecotype R108 was used as the WT plant in this study. The mutant lines of *MtLHY* and *MtLHY*-overexpressing plants were obtained from our previous work [10]. *M. truncatula* seedlings were initially cultured in artificial climate incubators at a 22 °C 16 h (daytime)/8 h (night) photoperiod cycle and 100 μmol·m^−2^·s^−1^ light, under 70 to 80% relative humidity. After two weeks of growth, similar seedlings were transplanted into pots (10 × 10 × 8 cm) filled with soil and Hoagland nutrient solution. The plants were then grown in a greenhouse under environmental conditions of 22 °C 16 h (daytime)/8 h (night) photoperiod cycle, 150 μmol·m^−2^·s^−1^ light, and 70 to 80% relative humidity. 

### 4.2. Salt Treatments and Sampling 

After being cultivated in the greenhouse for 2 weeks, 4-week-old plants were subjected to a salt stress test by irrigating them with 20 mL of 150 mM NaCl solution every 3 days for 4 weeks. To conduct transcriptional analysis, leaves were collected at 0 h, 3 h, 9 h, 12 h, and 24 h on the first day with or without 150 mM NaCl treatment. For physiological measurements, leaves and roots were sampled at 7th days. 

### 4.3. Measurements of Survival Rate and Physiological Index

After 150 mM NaCl treatment, the survival rate statistics of plants were determined according to the performance of leaves. Plants with at least five green trifoliate leaves were considered to have survived. The experiments were performed on at least 3 independent biological replicates, and each replicate included 15 plants. The MDA content was determined as previously reported [44]. H_2_O_2_ accumulation was measured using the Hydrogen Peroxide Assay Kit (S0038, Beyotime, Haimen, China) to determine the H_2_O_2_ content. For Na^+^ and K^+^ concentrations, samples were heated at 200 °C for 8 h, and their contents were measured using an inductively coupled plasma optical emission spectrometer (ICAP6300). SOD activity was determined using the Total Superoxide Dismutase Assay Kit with WST-8 (S0101S, Beyotime, China). To measure chlorophyll content, fresh leaves were ground and transferred to an extract solution (80% acetone with 1 μM KOH), and the total chlorophyll content was measured using a UV–Vis spectrophotometer. 

### 4.4. Histochemical Detection of ROS

The root samples were subjected to DAB staining by immersing them in a 1 mg/mL DAB solution for 8 h at 22 ℃ in the dark. After that, the samples were treated with 95% ethanol and boiled to remove excess stains. Similarly, for NBT staining, the root samples were immersed in NBT stain solution for 3 h at 22 ℃ in the dark, followed by treatment with 95% ethanol and boiling to decolorize. The samples were then cooled and transferred to 75% ethanol before being observed under a fluorescence microscope (Olympus, Tokyo, Japan).

### 4.5. RNA Extraction and RT-qPCR Analysis

Total RNA was extracted from leaves using RNAiso Plus (TaKaRa, Tokyo, Japan). The plant materials were ground into a fine powder using the Tissuelyser-48 (Shanghai Jingxin, Shanghai, China). Three biological samples were collected, and RT-qPCR analysis was conducted as described previously [45]. The UBQ gene was utilized as the internal reference, and all primers used are listed in Supplemental Table S1. 

### 4.6. DPBA Staining

The samples were stained with DPBA and gently rotated for 5 min, followed by careful washing with H_2_O to remove the DPBA. The fluorescence signal was immediately detected using the LSM880 confocal laser scanning microscope (Zeiss, Oberkochen, Germany). The emission spectrum for Kaempferol-DPBA was set to 475–500 nm, while for Quercetin-DPBA, it was set to 585–619 nm. 

### 4.7. Flavonoid Content

The flavonoid content was evaluated through the use of liquid chromatography–tandem mass spectrometry (LC-MS), following previously established methods [46]. The relative content of flavonol was quantified with daidzin as an external standard, and the unit of measurement for the relative content is presented as ng/g dry weight. Three biological replicates were obtained for LC-MS analysis. 

### 4.8. Luciferase Imaging Assay

The full-length coding sequence (CDS) of *MtLHY* was amplified by PCR and fused to pEarleyGate 103 plasmids as the effector. To create the reporter construct, a promoter fragment of approximately 2.5 kb upstream of *MtFLS* was PCR-amplified and cloned into the pGreenII-0800-LUC vector. These constructs were then introduced into A. tumefaciens GV3101, along with the pSoup19 helper plasmid, and coexpressed in 4-week-old N. benthamiana leaves. After 48 h, the infiltrated tobacco leaves were harvested and luciferase fluorescence signals were examined using a plant living imaging system (Tanon 5200, Shanghai, China) after being sprayed with Luciferin. The remaining leaves were then punched and powdered in liquid nitrogen to measure LUC and REN activity using a dual-luciferase reporter (DLR) assay system (Promega, Madison, WI, USA) on the Centro XS LB960 (Berthold, Schwarzwald, Germany). The ratio of LUC to REN (LUC/REN) was used to demonstrate the activity of transactivation. The primer sequences are listed in Supplemental Table S1. 

### 4.9. Yeast One-Hybrid (Y1H) Assay

The yeast one-hybrid assays were conducted using the Matchmaker Gold Yeast One-Hybrid System (Clontech, Mountain View, CA, USA). The full-length CDS of *MtLHY* was amplified by PCR and fused to the pGADT7 vector to create the pGADT7-*MtLHY* prey. The DNA fragment from the *MtFLS* promoter was amplified and inserted into the pAbAi vector to generate the pFLS-pAbAi bait. The pFLS-pAbAi vector was linearized and cotransformed with pGADT7-*MtLHY* into the Y1H Gold yeast strain. Transformants were selected on SD/-Leu/-Ura/AbA media using aureobasidin A (AbA). The primer sequences can be found in Supplemental Table S1. 

### 4.10. Statistical Analysis

Most of the pairwise comparisons between the means were performed using a two-sided Student’s *t*-test method (* *p* < 0.05, ** *p* < 0.01, *** *p* < 0.001) with GraphPad Prism version 9.0 software. The statistical comparison of various experimental groups and the control was conducted by a one-way ANOVA tool based on the Dunnett test (* *p* < 0.05, ** *p* < 0.01, *** *p* < 0.001).

## 5. Conclusions

In this study, our data indicate that the core circadian clock component, *MtLHY*, plays a positive role in the response to salt stress in *M. truncatula*. Our results demonstrate that loss-of-function mutants of *MtLHY* were more sensitive to salt stress treatment, while *MtLHY*-overexpressing lines exhibited increased salt stress tolerance. Based on the physiological indices obtained under salt treatment, it is likely that *MtLHY* plays a crucial role in the response to salt stress by regulating ROS and Na^+^/K^+^ homeostasis. Additionally, overexpression of *MtLHY* enhances salt stress tolerance by increasing flavonol accumulation through the regulation of flavonol synthase gene expression. Furthermore, the application of exogenous flavonols improved salt stress tolerance in *M. truncatula*. Therefore, our study has identified the important roles of *MtLHY* in the response to salt stress, which links the circadian clock with flavonoid biosynthesis in *M. truncatula*. 

## Data Availability

The data presented in this study are available in supplementary material here.

## Supplementary Materials

The supporting information can be downloaded at: https://www.mdpi.com/article/10.3390/ijms24129948/s1.
