# Supplementary material for: Late Elongated Hypocotyl Positively Regulates Salt Stress Tolerance in Medicago truncatula
Source: Int J Mol Sci. 2023 Jun 9;24(12):9948. doi: 10.3390/ijms24129948 (PMC10298455; doi:10.3390/ijms24129948)
Supplement: Supplementary file 1 [file ijms-24-09948-s001.zip › ijms-2398909-supplementary.pdf]

Supplementary Table S1. List of primers used in this study.

MtLHY-CDS-F	5′-ATGGATGCAGCAGCATACTCCTCTG-3′
MtLHY-CDS-R	5′-AATAGAAGTCCCCCCTTCCAGG-3′
MtLHY-CDS-R1	5′-CTTGATTGCCGAAGATACAGA-3′
MtLHY-CDS-F2	5′-AAAAGCAGGCTCCGCGATGGATGCAGCAGCATACTCCTCTG-3′
MtLHY-CDS-R2	5′-AGCTGGGTCGGCGCGAATAGAAGTCCCCCCTTCCAGG-3′
MtLHY-qRT-F	5′-CTGTGCACGTTGTTGATGGG-3′
MtLHY-qRT-R	5′-GCAGCTGGATTTGCGAAGAG-3′
MtFLS-qRT-F	5′-GCTGCAGGTGGAGATGAGTT-3′
MtFLS-qRT-R	5′-TGAAGGCCCTGCACTTCATT-3′
MtFLS-PROMOTER-F1	5′-TCTATCTCAACTACCCACCAA-3′
MtFLS-PROMOTER-R1	5′-TGTTACTTTGGTTTTTTTCTTTCT-3′
MtFLS-PROMOTER-F2	5′-ATAAGCTTACCTCCAGATTTGTAGGAACGGCTC-3′
MtFLS-PROMOTER-R2	5′-ATAGAGCTCGTGAGTATGAGAGAGAAAGTTGTCA-3′
Medtr1g097910-qRT-F	5′-GTGCCCGTGTGTTGGTTGTC-3′
Medtr1g097910-qRT-R	5′-CTGTCCAACAAGGCTGTCCA-3′
Medtr2g058470-qRT-F	5′-CCTGGTGCCGATTACCAACT-3′
Medtr2g058470-qRT-R	5′-CCACCTGCAAAACACCCTTG-3′
Medtr7g016820-qRT-F	5′-CACTGCTGTCACATTTCGTG-3′
Medtr7g016820-qRT-R	5′-GGTATTGGGTCAGAGCCAAC-3′
Medtr1g115840-qRT-F	5′-CACCAGGGACTGCTGCTTTC-3′
Medtr1g115840-qRT-R	5′-GTCTCCAACACCGCCTCCGA-3′
Medtr1g115870-qRT-F	5′-CCGTTGACGGGACACCAATA-3′
Medtr1g115870-qRT-R	5′-AGGATAGAGGAGCCTGGTGG-3′
Medtr3g058610-qRT-F	5′-AACAAGTGGGTGGCCTTCAA-3′
Medtr3g058610-qRT-R	5′-CTGGAATTCACCACTGCCCT-3′
Medtr4g109470-qRT-F	5′-ACTCCTCTATCCCTCCCACG-3′
Medtr4g109470-qRT-R	5′-TTAGGGTCGCGTGATATGGC-3′
Medtr4g088160-qRT-F	5′-CTACTGCAGGAATGGCCACA-3′
Medtr4g088160-qRT-R	5′-CGACACCAGCTCTTGTGAGT-3′
Medtr1g105020-qRT-F	5′-TGGTGGTGATACTTCAGGTGC-3′
Medtr1g105020-qRT-R	5′-ACCGGCTTCGAACTCCAAAA-3′
